# Curious creatures: a multi-taxa investigation of responses to novelty in a zoo environment

**DOI:** 10.7717/peerj.4454

**Published:** 2018-03-08

**Authors:** Belinda A. Hall, Vicky Melfi, Alicia Burns, David M. McGill, Rebecca E. Doyle

**Affiliations:** 1 Animal Welfare Science Centre, University of Melbourne, Melbourne, VIC, Australia; 2 Taronga Conservation Society Australia, Taronga Zoo, Sydney, NSW, Australia; 3 School of Life and Environmental Sciences, University of Sydney, Sydney, NSW, Australia; 4 Faculty of Veterinary and Agricultural Science, University of Melbourne, Melbourne, Australia

**Keywords:** Boldness, Novelty, Behaviour, Exploration, Personality, Welfare

## Abstract

The personality trait of curiosity has been shown to increase welfare in humans. If this positive welfare effect is also true for non-humans, animals with high levels of curiosity may be able to cope better with stressful situations than their conspecifics. Before discoveries can be made regarding the effect of curiosity on an animal’s ability to cope in their environment, a way of measuring curiosity across species in different environments must be created to standardise testing. To determine the suitability of novel objects in testing curiosity, species from different evolutionary backgrounds with sufficient sample sizes were chosen. Barbary sheep (*Ammotragus lervia) n* = 12, little penguins (*Eudyptula minor) n* = 10, ringtail lemurs (*Lemur catta) n* = 8*,* red tailed black cockatoos (*Calyptorhynchus banksia) n* = 7, Indian star tortoises (*Geochelone elegans) n* = 5 and red kangaroos (*Macropus rufus) n* = 5 were presented with a stationary object, a moving object and a mirror. Having objects with different characteristics increased the likelihood individuals would find at least one motivating. Conspecifics were all assessed simultaneously for time to first orientate towards object (s), latency to make contact (s), frequency of interactions, and total duration of interaction (s). Differences in curiosity were recorded in four of the six species; the Barbary sheep and red tailed black cockatoos did not interact with the novel objects suggesting either a low level of curiosity or that the objects were not motivating for these animals. Variation in curiosity was seen between and within species in terms of which objects they interacted with and how long they spent with the objects. This was determined by the speed in which they interacted, and the duration of interest. By using the measure of curiosity towards novel objects with varying characteristics across a range of zoo species, we can see evidence of evolutionary, husbandry and individual influences on their response. Further work to obtain data on multiple captive populations of a single species using a standardised method could uncover factors that nurture the development of curiosity. In doing so, it would be possible to isolate and modify sub-optimal husbandry practices to improve welfare in the zoo environment.

## Introduction

Individuals in a single population will often have different behavioural responses when faced with the same conditions (Coleman, 2012; Mehta & Gosling, 2008). Individual variation has been flagged as an important factor for captive animal welfare, as success both in captivity and in the wild comes down to individual coping styles (Koolhaas et al., 1999; McDougall et al., 2006; Watters & Powell, 2012). Determining the traits that allow animals to adapt to life in captivity is difficult, as multiple factors from both an evolutionary level and an individual level are at play. Innate drives common to all within a species can be difficult to accommodate for in captivity and the inability to express this normal behaviour can lead to increased stress (Mason, 2010). Variation in individual traits such as boldness and previous experience further influence how an individual can manage challenges faced in captivity (Franks et al., 2013; Stoinski, Jaicks & Drayton, 2012; Tetley & O’Hara, 2012). To be able to assess how different species manage life in captivity, it is important to identify traits that are shared between animals that thrive in a captive setting and then create an efficient and effective way to detect these traits both between and within species. Parallels between coping with captive life and “human-induced rapid environmental change” in the wild have already been made, suggesting that species which exhibit high behavioural plasticity in the wild are also able to cope well with captive housing with the inverse also being true (Mason et al., 2013).

Curiosity has been described as a driving force behind increased interaction with one’s environment (Lilley, Kuczaj & Yeater, 2017). In humans, curiosity has been linked to an increased perception of positive outcomes (Maner & Gerend, 2007), decrease in depression (Kaczmarek et al., 2014), improved psychological and emotional wellbeing (Wang & Li, 2015) and an enhanced ability to deal with distress (Denneson et al., 2017). It can be argued that, as many species are known to share neurological similarities with humans, such as in the case of chimpanzees and their resting brain activity (Rilling et al., 2007) and vocalization interpretation in dogs (Andics et al., 2014), similar functions could also be shared with other species. Within the published literature on animal behaviour, curiosity is often referred to as “exploratory behaviour ” or “coping style” (Dingemanse et al., 2002; Murphy, 1978). Multiple species have been successfully assessed for curiosity using their behavioural responses to novel objects (Coleman, Tully & McMillan, 2005; Fox & Millam, 2007; Frost et al., 2007; Glickman & Sroges, 1966; Powell & Svoke, 2008; Stöwe et al., 2006; Svartberg, 2005). As curiosity is a trait found in many species, the relationship between high levels of curiosity and positive welfare might be found in species other than humans. Some evidence exists in the field of animal behaviour to suggest a positive relationship exists between the bold-shy continuum and stress axis in zebra fish (*Danio rerio*) (Oswald et al., 2012), further supporting this idea.

Powell & Svoke (2008) suggested that curiosity towards novel objects could be used as a personality assessment tool, a technique that has been shown to be successful in dogs (Svartberg, 2005) and in horses using open field tests (Napolitano et al., 2008). Curiosity can be assessed by observing an animal’s aversion or attraction to a novel sound, smell or object, as well as assessing other behaviour alterations displayed while exposed to the novel stimulus (Powell & Svoke, 2008). For example, animals that are fearful, shy or not inquisitive will often show an increased latency to approach a new object (Forkman et al., 2007). For information on curiosity to be obtained, novelty tests need to be designed with the specific species in mind. Modifying objects to better suit a species’ proclivities improves the chances that the animals will be motivated to interact with it and reduces potential negative responses such as anxiety (Heyser & Chemero, 2012). To do this, the anatomical, behavioural, social and physiological traits of a species should be considered when choosing novel objects (Goodrick, 1973; Heyser & Chemero, 2012). Differences in species perceptions can cause a seemingly non-threatening novel object to be fear-provoking to some species (Gray, 1987), therefore not giving accurate measures of curiosity. Events that are unexpected, such as objects that move and situations that are unpredictable, have been shown to elicit fear in some animals (Boissy & Bouissou, 1995) and may not always be approached if the level of fear outweighs the desire to investigate. Similarly, when used in animals without self-recognition, mirrors can provoke aggressive responses (Balzarini et al., 2014).

The aim of this study was to assess which types of novelty tests would be suitable to assess curiosity in a variety of zoo species. Here we use the following variables to determine the level of curiosity: latency to first orient towards the object, latency to make contact, frequency of interaction, and total duration of interaction with novel objects. Barbary sheep (*Ammotragus lervia)*, little penguins (*Eudyptula minor)*, ringtail lemurs (*Lemur catta),* red tailed black cockatoos (*Calyptorhynchus banksia)*, Indian star tortoises (*Geochelone elegans)* and red kangaroos (*Macropus rufus)* were selected as there is currently a lack of research in curiosity involving these species, and there were sufficient sample sizes available for testing within the zoo. Having such a diverse range of species also ensures representation of animals with an assortment of sensory and behavioural differences.

## Methods

This study took place from October 2015–January 2016 at Taronga Zoo in Sydney, Australia. A total of 44 individuals from six species were observed in this study. Details on the species and individuals are presented in Table 1.

**Table 1:** Taxa signalment, enclosure type, enrichment provided, and summary of novel object performance in accordance with “characteristics of an effective test” (see “novel objects” in methods).

Taxa	Common name	M:F:Juveniles	Age range	Enclosure type	Previous enrichment	Stationary	Moving	Mirror
*Ammotragus lervia*	Barbary sheep	7:5:0	4–12 years	Closed^a^	-Browse -Substrate changes -Scent sprays -Food Smears -Ice blocks in summer	Ineffective	Ineffective	Ineffective
*Calyptorhynchus banksia*	Red tailed black cockatoos	4:3:0	Unknown	Closed^a^	-Seed balls /pinecones -Nesting material -Papermache food balls	Ineffective	Ineffective	Ineffective
*Geochelone elegans*	Indian star tortoise	3:2:0	15–19 years	Closed^a^	-Substrate changes	Ineffective	Ineffective	Effective
*Eudyptula minor*	Little penguins	0:2:8 (Data not used on 3 of the juvenile males due to moulting)	1–12 years	Closed/visitor interactions^b^	-Frozen fish ice blocks -Bubbles/water streams -Training with keepers for cognitive tasks -Daily visitor hand feeding	Ineffective	Ineffective	Effective
*Macropus rufus*	Red kangaroos	0:4:1	10 months– 9 years	Walk through^c^	-Browse -Substrate changes -Change of enclosure set up	Effective	Effective	Effective
*Lemur catta*	Ringtail lemurs	8:0:0	6–16 years	Walk through^c^	-Browse -Smear boards -Puzzle feeders/kongs -Scent sprays	Effective	Effective	Effective

**Notes.**

a Closed enclosure—animals are enclosed and have no close contact with visitors.

b Visitor interactions—visitors can interact with the animals for short periods while supervised by keepers.

c Walk through—animals enclosed in an enclosure with airlocks, allowing for visitors to walk along a path through the enclosure. Interactions between animals and visitors are always monitored.

Animals were assessed for their response to novelty at the individual level, however in order to minimise disturbance, they were kept in their usual social groups, and presented with the objects as a group within their usual enclosures. Individual Barbary sheep and little penguins were identified using existing visual tags, red kangaroos, ringtail lemurs and red tailed black cockatoos were identified by discernible physical features, and star tortoises were identified using small coloured marks on their carapace. Due to three individuals moulting during the experimental period, data was collected from only seven of the 10 penguins housed in the enclosure.

This study was approved by the Taronga Conservation Society and was conducted in accordance with the Exhibited Animals Protection Act 1986.

## Materials

Characteristics of an effective novel object test were identified as being: (1) the ability to identify individual differences within the group; (2) have objects that appeal to the physical capabilities in a range of species; (3) generate a novel response, without eliciting high levels of visible stress (fleeing, erratic behaviour, aggression) or disrupting routines. Three different novel objects were used to fit these criteria: a stationary object, a moving object and a mirror. Three objects with different physical characteristics were chosen to increase the chances that animals with different sensitivities and interests would be motivated to investigate at least one of them. All objects were visual in nature as other sensors would be difficult to measure. The sizes of the stationary and moving objects were scaled to approximately one third of the average height of the species to control for the size differences between the species. The mirror was scaled down for the tortoises only as the larger mirror could not fit in the enclosure.

**Figure 1:**
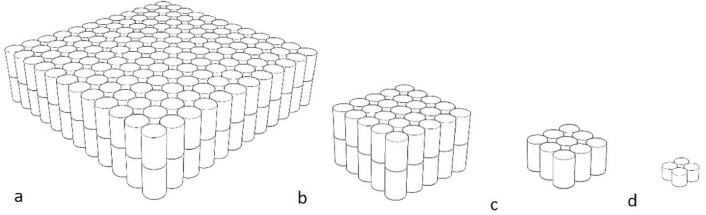
Diagrams of stationary objects. (A) 33 × 33 × 9 cm (B) 14.5 × 14.5 × 9 cm (C) 8.5 × 8.5 × 4.5 cm (D) 4.5 × 4.5 × 2.2 cm. All objects were fluorescent orange.

The stationary objects, made by the primary researcher, were solid fluorescent orange rectangular prisms made from non-toxic recycled cork, which was soft enough to not damage either the animals or the enclosure. The softness of the object was a consideration for future work where particularly strong animals, like great apes, may throw it. The colour was chosen as it was not routinely found in the natural environment and for dichromats, the fluorescence would be different from the surrounding environment. It has been shown in horses (known dichromats), that they are able to discern differences in colour based on brightness (Geisbauer et al., 2004). Objects in four different sizes were created, with the diameters being 33 × 33 × 9 cm (Barbary sheep, red kangaroos), 14.5 × 14.5 × 9 cm (ringtail lemurs, red tailed black cockatoos), 8.5 × 8.5 × 4.5 cm (little penguins) and 4.5 × 4.5 × 2.2 cm (star tortoises) (Fig. 1). This object was designed to be the most benign as it was scaled to be smaller than the animals and stationary.

**Figure 2:**
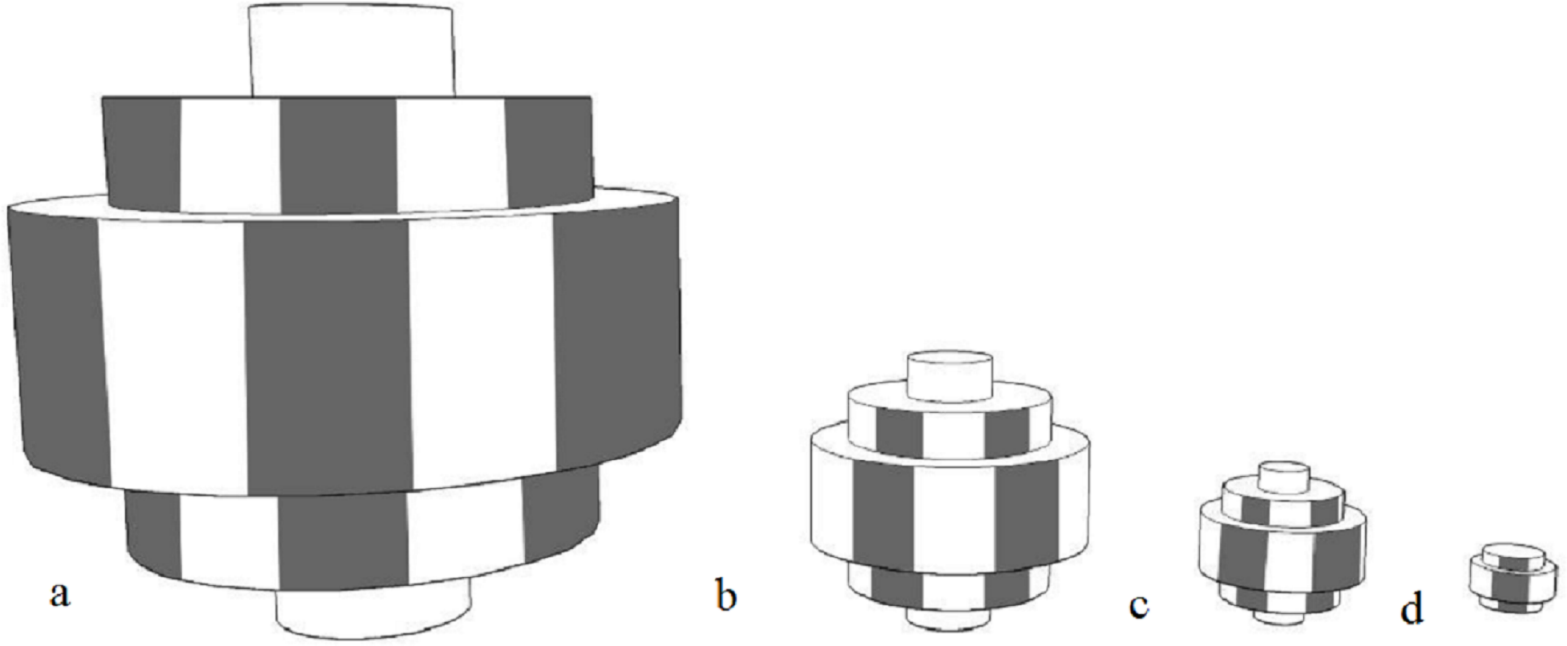
Diagram of moving objects. (A) 33 × 33 × 33 cm (B) 14.5 × 14.5 × 14.5 cm (C) 8.5 × 8.5 × 8.5 cm (D) 4.5 × 4.5 × 4.5 cm.

The moving objects, made by the primary researcher, were black and white striped stepped toroidal shapes made by layering expanded polypropylene foam (non-toxic and soft). As with fluorescence, sharp recurring lines are not often found in the natural environment, and the appearance of black and white should not be altered too much in the event of differing visual capabilities. A rope through a hole in the centre of the object was used to move it. The moving object was intended to attract animals that are more stimulated by movement or that are bold enough to overcome the uncertainty of the movement to investigate. Objects in four different sizes were created with the objects measuring 33 × 33 × 33 cm (Barbary sheep, red kangaroos) 14.5 × 14.5 × 14.5 cm (ringtail lemurs, red tailed black cockatoos) 8.5 × 8.5 × 8.5 cm (little penguins) 4.5 × 4.5 × 4.5 cm (star tortoises) (Fig. 2).

A 1 × 1.5 m silver acrylic mirror was used for the Barbary sheep, little penguins, ringtail lemurs*,* red tailed black cockatoos and red kangaroos. Two small circular mirrors of 11.5 cm diameter were used for the star tortoises. The reflective surface of the mirror allows for a feedback element that the other two objects lacked, adding a different characteristic to the test; possibly attractive to social animals.

## Procedure

Novel object testing was performed over three consecutive days with a different object being tested each day. The order in which the objects were presented was the same for each species (stationary object, moving object, mirror). On each day, the object was placed in the enclosure within sight of the normal feeding area by a keeper or experimenter depending on availability. Testing was performed before the zoo opened to ensure that responses were not influenced by the presence of visitors. As this is a feeding time, the animals were all present except for one case. Due to large enclosure size, only three of the five kangaroos were present for the first stationary object test. To rectify this problem, footage from the first test was used for the three individuals present and then the stationary object was represented on a separate day when the two missing individuals were present and the others were not. Reactions were recorded for the two missing individuals during the second test. Testing began once the keeper had left the enclosure, and ran for 15 min before the objects were removed. The moving object was manually moved, by the experimenter from outside the enclosure. The object was moved approximately 5 cm every 60 s by pulling the rope attached to it. Each object was presented only once to control for the possibility of habituation.

Conspecifics were assessed at the same time (with the exception of the stationary object in the red kangaroos) and behaviours of all individuals were digitally recorded for 15 min and analysed for the following: time to orient towards object (s), latency to make contact (s), frequency of interaction and total duration of interaction (s). In cases where an individual did not make physical contact with the object, a minimum distance in body lengths was estimated. The maximum estimate for this was four body lengths. Interaction with both the stationary and moving object was defined as when the animal made physical contact with the object, whereas interaction with the mirror included contact as well as the animal following its own movements in the mirror without contact.

## Data Analysis

Means and standard deviations were calculated for latency to contact (s), time to orient (s) and duration of interaction (s) for each species during each of the three novel object tests. Graphical representations of the ‘time to orient’, ‘latency to contact’ and ‘duration of interaction’ were used to compare differences between species and the tests. Qualitative descriptions of the observations and comparisons between species were the primary reporting method used in this study and the graphical representation with summary statistics helps support these.

## Results

**Figure 3:**
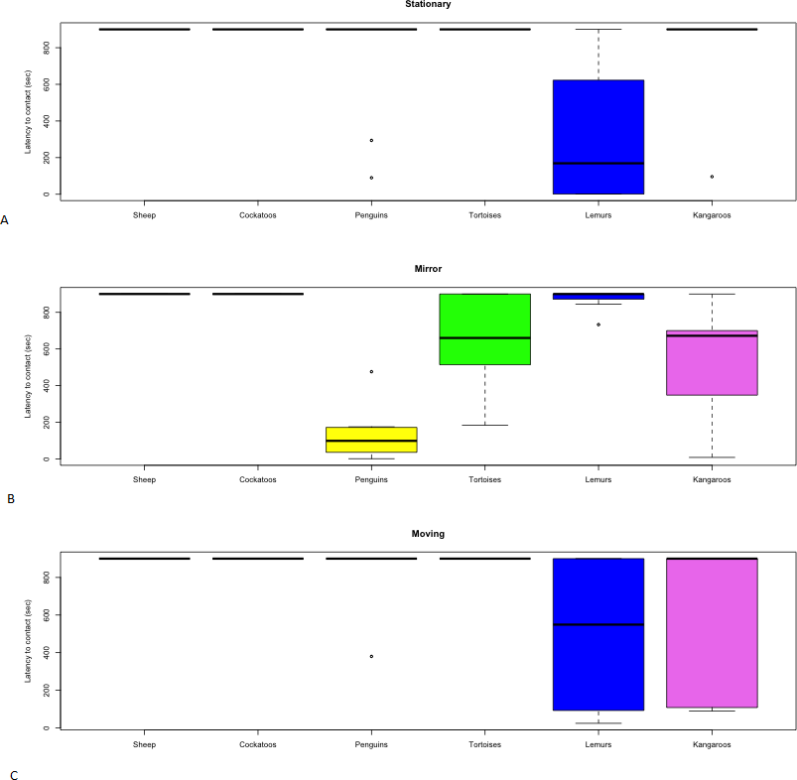
Distribution of latency to contact novel objects. (A) Stationary, (B) Mirror, (C) Moving by species.

**Figure 4:**
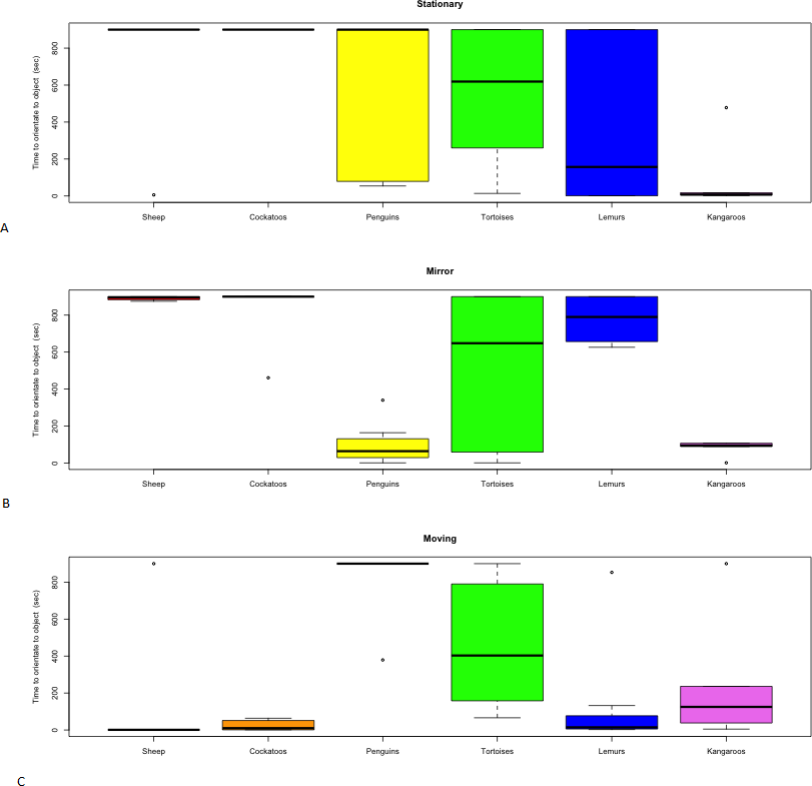
Distribution of time to orient towards novel objects. (A) Stationary, (B) Mirror, (C) Moving by species.

The results of the novel object tests for all six species are listed in Table 2 and are presented in Figs. 3 and 4. The objects effectiveness, in accordance to the characteristics of an effective test identified in the methodology, are displayed for each species in Table 1.

**Table 2:** Taxa responses to the three novel object tests; mean and standard deviations presented in parentheses.

Taxa	Novel object^a^	Time to orient (s)	Latency to contact (s)	Duration of interaction (s)	Number to approach^b^	Body lengths from object^c^
Barbary sheep	Stationary	825.5 (258.1)	900 (0)	0 (0)	0	+4 (0)
	Moving	75.9 (259.5)	900 (0)	0 (0)	0	+4 (0)
	Mirror	891.8 (10.7)	900 (0)	0 (0)	0	+4 (0)
Indian star tortoise	Stationary	538.6 (393.9)	900 (0)	0 (0)	0	3.6 (0.9)
	Moving	463.4 (371.5)	900 (0)	0 (0)	0	2.7 (1.8)
	Mirror	501.6 (443.1)	631.4 (300.0)	85.0 (80.9)	3	1.6 (2.2)
Little penguins	Stationary	544.7 (443.2)	697.7 (350.5)	1.6 (2.8)	2	2.4 (2.1)
	Moving	900 (0)	900 (0)	0 (0)	0	+4 (0)
	Mirror	130.6 (106.3)	174.0 (146.9)	202.0 (133.6)	7	0 (0)
Red kangaroo	Stationary	102.0 (210.3)	739.2 (359.6)	1.0 (2.2)	1	2.2 (1.8)
	Moving	260.8 (368.3)	579.6 (438.8)	1.2 (1.8)	2	2.4 (2.2)
	Mirror	80.0 (44.9)	525.6 (350.5)	95.2 (66.6)	4	0.8 (1.8)
Red tailed black cockatoo	Stationary	900 (0)	900 (0)	0 (0)	0	+4 (0)
	Moving	25.7 (28.6)	900 (0)	0 (0)	0	3.7 (0.8)
	Mirror	837.3 (165.9)	900 (0)	0 (0)	0	+4 (0)
Ringtail lemur	Stationary	307.8 (384.7)	380.1 (436.7)	7.0 (6.2)	5	1.3 (1.8)
	Moving	131.1 (294.8)	500.9 (430.8)	0.8 (0.9)	4	1.1 (1.8)
	Mirror	777.5 (132.0)	872.1 (59.5)	58.9 (79.9)	2	2.4 (1.8)

**Notes.**

a Novel object presented.

b The number of animals to contact/interact with the novel object.

c A maximum distance of four body lengths could be estimated; a score of +4 indicates that no animals approached the novel object.

### Stationary object

The stationary object elicited a range of responses between species. Neither the Barbary sheep nor the red tailed black cockatoos approached or interacted with the stationary object and variation in individual responses was negligible (Fig. 3). The time it took for the Indian tortoises to orient towards the stationary object varied but no contact was made (Figs. 4 and 3), and of the three penguins that orientated towards the stationary object, two made contact for a brief period. In contrast, the red kangaroos varied between individuals in their response to the stationary object, differing in both time to orient and time to make contact (Figs. 4 and 3). On average the ring-tailed lemurs approached the stationary object the fastest out of the three objects; however, as with the kangaroos, individuals within the group varied in their response time (Fig. 3).

### Moving object

Barbary sheep were slower to orient towards both the stationary object and mirror compared to the moving object (Fig. 4), though no physical contact was attempted (Fig. 3). The red tailed black cockatoos also displayed a faster orientation time to the moving object compared to the other objects. Still, none of the cockatoos made contact with the object (Fig. 3). The tortoises showed individual variation in orientation time towards the moving object (Fig. 4), with only one individual moving towards the object; however, no contact was made. The other tortoises made no movement towards the moving object from their original positions. The moving object caused the penguins to remain in the water making individual identification and orientation impossible to measure. No penguins approached and all maintained a large distance from the object. Both the kangaroos and lemurs showed individual variation in their interaction and orientation towards the moving object (Figs. 3 and 4).

### Mirror

As with the stationary object, the Barbary sheep and the red tailed black cockatoos showed no approach or interaction with the mirror (Fig. 3). The time it took the tortoises to orientate towards the mirror varied (Fig. 4). Three out of five of the tortoises contacted the mirror, but none engaged with the other two novel objects. The mirror elicited a faster orientation for the little penguins (Fig. 4) compared to the other two objects, a shorter latency to approach the mirror as well as a longer duration of interaction. For the kangaroos, the mirror test yielded longer interaction times, compared to the other two objects (Table 2). The ringtail lemurs however, were slower to orientate to the mirror compared to the other two objects, and only two of the eight lemurs interacted with the mirror. Despite this, the duration of interaction with the mirror was much longer than the stationary or moving objects (Table 2) even though only one lemur made physical contact with the mirror surface.

## Discussion

Using the three novel objects, the individual behavioural responses of six different species were evaluated to ascertain if it was possible to: (1) identify individual differences within the group; (2) motivate animals to notice and possibly interact with objects; (3) suit the physical capabilities of a range of species without eliciting high levels of visible stress (fleeing, erratic behaviour, aggression) or disrupting routines. By addressing these three criteria for the novel objects we decrease the likelihood that animals which do not interact with any of the objects do so due to a low level of curiosity and not due to the object being irrelevant to the species or due to fear. Using the three objects, individual variation in response to the objects within the normal enclosure was seen for four of the six species tested (little penguins, ring-tailed lemurs, Indian star tortoises and red kangaroos). For the red tailed black cockatoos and Barbary sheep, none of the novel objects elicited any interaction so individual variation could not be assessed. The variation in response between and within species shows how important it is to have multiple objects with varied characteristics to be able to correctly identify curiosity in animals across different species.

In studies involving animal motivation and response such as this one, evolutionary and ecological variation is important. The Barbary sheep and the red-tailed black cockatoos are the two species which displayed avoidance behaviours with all three objects. While these species are very different they do share a few similar ecological traits, which may have contributed to the similarity in responses. In wild conditions Barbary sheep have large home ranges (Hampy, 1978) as do red-tailed black cockatoos (Garnett & Franklin, 2014); however, in most zoos, the size of their enclosures are a fraction of the size of the natural range. Ungulates have been found to exhibit increased vigilance and fear towards external stimuli when habitat cover is decreased (Underwood, 1982a), when herd sizes are small (Underwood, 1982b) and when experiencing chronic stress such as repeated adverse handling (Destrez et al., 2013). Studies looking at exploration and neophobia in parrots show that seed eating parrots have longer latencies to explore novel objects than bud-eating parrots suggesting that evolving to suit their ecological niche has trade-offs in how they perceive their environment (Mettke-Hofmann, Winkler & Leisler, 2002). Other potential ecological pressures were also seen with the little penguins. Penguins displayed land-avoidance when presented with the moving object, but not with the stationary object, suggesting that the moving object was fear-provoking for the penguins. The moving object was seen only from a distance while the penguins were floating on the water’s surface, so it is possible that its form could not be interpreted correctly as penguins are said to be near sighted on land and far sighted in water (Sivak & Millodot, 1977). However, it would serve the penguins well to be cautious of moving objects on land as they have native land predators such as sea birds and reptiles as well as introduced species such as cats, dogs and foxes (Stahel, Gales & Burrell, 1987). Both the lemurs and kangaroos showed quick interactions with all three objects. These species both have an ability to adapt to harsh environmental conditions in the wild (Gould, Sussman & Sauther, 1999; Sharman & Pilton, 1964) so curiosity towards novel food items and environments would be an advantage for both species.

The sociability of a species may also influence how an animal responds to a novel object. Social interactions could affect an animals response to novel objects in more complex ways than just the presence or absence of other individuals alone. As most penguins in the current study only interacted with the mirror, it seems likely that the reflective properties of the object appealed to them and this characteristic would be the most likely to elicit a curious response from the species. The mirror’s appeal may hinge on the fact that little penguins are a highly social species where individuals travel between colonies and interact with unfamiliar conspecifics often (Reilly & Cullen, 1982). It is not possible to ascertain if social interaction factored into their interest as social behaviours towards the reflected images were not assessed. Further research into this possibility would shed light on what properties of the mirror were most appealing to the penguins. The ring-tailed lemurs, also social animals (Nakamichi & Koyama, 1997), did not show the same level of interest in the mirror as the penguins. The lack of interest seen is this study is contradictory to previous research which found that ring tailed lemurs prefer mirrored surfaces to standard surfaces even after repeated exposure (Fornasieri, Roeder & Anderson, 1990). As no evidence of self-recognition has been reported in ring-tailed lemurs (Inoue-Nakamura, 1997), it is likely reflected images would be perceived as a foreign individual. Interestingly there were no signs of aggression towards the image, which would be expected when males encounter males from other groups (Nakamichi & Koyama, 1997). Perhaps because of a lack of other sensory indicators such as scent, sound and touch, the reflected image was perceived as more perplexing than threatening. In contrast the star tortoises, who showed a preference for the mirror objects are not known for their social skills. Not only did they prefer the mirrors but once one tortoise approached the mirror, others came to join from areas of the enclosure which were not in direct line of sight. It is difficult to tell if the first individual to interact with the mirrors affected the response of the others. There is evidence that there may be some aspect of social learning among red-footed tortoises *Chelonoidis carbonaria*, (Wilkinson et al., 2010a), as well as the ability to follow gaze direction of conspecifics (Wilkinson et al., 2010b), so a social effect might have been involved in the widespread response observed in this study.

In addition to ecological factors, previous life experience is also likely to influence an animal’s interactions with their environment. The cockatoos used in this study are provided with minimal “unnatural” materials in terms of housing, substrates or enrichment. This lack of previous experience with novelty and variation could explain the heightened neophobic reactions observed as environmental enrichment has been suggested to manage neophobia in parrots (Fox & Millam, 2004). Similarly, negative past experiences have been found to contribute to increased fear in sheep (Dwyer, 2004). The lemurs and kangaroos used in this study are both housed in “walk though” enclosures which affords them with more novel simulation through indirect interactions with visitors. From the results of this study alone, it is not possible to say whether the species propensity for curiosity is nurtured by this type of housing or if evolutionary history affords the ring-tailed lemurs and red kangaroos to be more curious and less fearful. Reduced life experience may also influence curiosity as shown by the increased time the juvenile kangaroo spent with the mirror object compared to the adults in the group. Juveniles have been found to have increased curiosity towards novelty compared to adults in rats *Rattus norvegicus* (Douglas, Varlinskaya & Spear, 2003), neotropical raptors *Milvago chimango* (Biondi, Bó & Vassallo, 2010) and vervet monkeys *Chlorocebus pygerythrus* (Fairbanks, 1993). Increased curiosity during early life may be an important aspect of learning, as critical learning periods occur during this time (Knudsen, 2004). Further investigation involving different populations of the same species with individuals of various ages housed in different enclosure conditions would help determine if enclosure design and exposure to varied enrichment protocols helps promote curious behaviour and reduces hesitation to interact with novel objects, or if evolutionary history is most important.

Due to the myriad of potential factors contributing to an animal’s behavioural response it is easy to see how limited knowledge of a species makes creating appropriate object for testing curiosity difficult. For example, reptiles have historically been deemed as unintelligent. Even though recent studies have shed light on reptile navigation skills (Day, Ismail & Wilczynski, 2003; Wilkinson, Chan & Hall, 2007), social learning (Wilkinson et al., 2010a) and cognitive mapping (Lopez et al., 2001), we still know relatively little about them (Burghardt, 2013). It is probable that we are not appealing to the tortoises’ propensities with our current novel objects. However, the way the star tortoises in this study actively sought out and interacted with the mirrors adds to growing evidence that reptiles have more cognitive needs than they are often given credit for. In understanding a species umwelt, we not only increase the chance of selecting motivating objects but also increase the accuracy of our interpretation. For example, the fast orientation that the Barbary sheep showed towards the novel objects, especially the moving object, is a trait shared by other ungulates and domesticated ovids (Désiré et al., 2004). However, orientation may not be the best indication of attention in this species, as domestic sheep have a 290° field of vision (Kendrick, 2008), which makes it possible that the objects were sighted in the peripheral vision of the Barbary sheep before they orientated their body towards the objects making orientation times for this species inaccurate. Behavioural responses may also be affected by the location of the object in penguins as they are dichotomous on land and sea, the same objects placed under the water or on land may provide us with different results.

## Limitations

There were two main limitations in this study. The first being small samples sizes and gender bias. Due to the nature of captive animals many of the groups are small and of a single gender due to housing restrictions. Repeated studies using different populations would assist in reducing this issue however, in the zoo environment this is unavoidable. It must also be mentioned that there are many limitations when testing a group of animals together such as a lack of experimental control, differential access to the objects, reduced accuracy in large groups (Cronin et al., 2017) and social influences (Arakawa, 2006; Stöwe et al., 2006; Frost et al., 2007). However, evaluating animals in their everyday environment can reduce external modifiers such as unfamiliar environments which can reduce the validity of the results (Cronin et al., 2017; Richter, Garner & Würbel, 2009). Cronin and colleagues (2017) have identified the use of multiple identical objects placed around the testing area to try to mitigate some of the dominance concerns and vicinity issues faced in this study, as occurred in the stationary object tests in the red kangaroos and star tortoises, and this practice may be useful in further studies in this area. Additional information may also be obtained in future research by recording how many ways an individual tried to interact with the object; this may require extending the duration of the experiment.

## Conclusion

This study has demonstrated that the behavioural responses to novelty differed within and between species depending on the characteristics of the objects themselves. This is an important preliminary step in developing tests for curiosity across species as it shows that there is need for novel objects with a range of characteristics to allow for accurate assessment of curiosity. Even if one object promotes a curious response in all individuals within a population (such as the mirror with the penguins), additional objects that cause variation in individuals may show the effect of personal experience or personality on behaviour. Before implementing a test for curiosity, it would be advisable to identify multiple motivating stimuli within the species being studied to increase the chances of accurately capturing curiosity. This can be done by combining knowledge of both the sensory limitations of the species and the ecological niche they occupy in the wild, as well as studies like this one to identify motivating characteristics of objects. By identifying common factors, such as husbandry practices or ecological similarities, shared by individuals and species which display curious and well adjusted behaviours towards novelty we may be able to modify management practices to improve the lives of captive animals.

##  Supplemental Information

10.7717/peerj.4454/supp-1 Supplemental Information 1 Raw data

10.7717/peerj.4454/supp-2 Supplemental Information 2 Enclosure layout and novel object placement Layouts of enclosures and novel object placement. Objects identified as moving (M), stationary (S) and mirror (X). a) little penguins b) Barbary sheep c) star tortoises d) ring tailed lemurs e)red-tailed black cockatoos f) red kangaroos.

## Additional Information and Declarations

### Data Availability

The following information was supplied regarding data availability: The raw data have been provided as a Supplemental File.

## References

[ref-1] Andics A, Gácsi M, Faragó T, Kis A, Miklósi Á (2014). Voice-sensitive regions in the dog and human brain are revealed by comparative fMRI. Current Biology.

[ref-2] Arakawa H (2006). Changes in the pattern of exploratory behavior are associated with the emergence of social dominance relationships in male rats. Developmental Psychobiology.

[ref-3] Balzarini V, Taborsky M, Wanner S, Koch F, Frommen JG (2014). Mirror, mirror on the wall: the predictive value of mirror tests for measuring aggression in fish. Behavioral Ecology and Sociobiology.

[ref-4] Biondi LM, Bó MS, Vassallo AI (2010). Inter-individual and age differences in exploration, neophobia and problem-solving ability in a Neotropical raptor (Milvago chimango). Animal Cognition.

[ref-5] Boissy A, Bouissou MF (1995). Assessment of individual differences in behavioural reactions of heifers exposed to various fear-eliciting situations. Applied Animal Behaviour Science.

[ref-6] Burghardt GM (2013). Environmental enrichment and cognitive complexity in reptiles and amphibians: concepts, review, and implications for captive populations. Applied Animal Behaviour Science.

[ref-7] Coleman K (2012). Individual differences in temperament and behavioral management practices for nonhuman primates. Applied Animal Behaviour Science.

[ref-8] Coleman K, Tully LA, McMillan JL (2005). Temperament correlates with training success in adult rhesus macaques. American Journal of Primatology.

[ref-9] Cronin KA, Jacobson SL, Bonnie KE, Hopper LM (2017). Studying primate cognition in a social setting to improve validity and welfare: a literature review highlighting successful approaches. PeerJ.

[ref-10] Day LB, Ismail N, Wilczynski W (2003). Use of position and feature cues in discrimination learning by the whiptail lizard (Cnemidophorus inornatus). Journal of Comparative Psychology.

[ref-11] Denneson LM, Smolenski DJ, Bush NE, Dobscha SK (2017). Curiosity improves coping efficacy and reduces suicidal ideation severity among military veterans at risk for suicide. Psychiatry Research.

[ref-12] Désiré L, Veissier I, Després G, Boissy A (2004). On the way to assess emotions in animals: do lambs (Ovis aries) evaluate an event through its suddenness, novelty, or unpredictability?. Journal of Comparative Psychology.

[ref-13] Destrez A, Deiss V, Leterrier C, Boivin X, Boissy A (2013). Long-term exposure to unpredictable and uncontrollable aversive events alters fearfulness in sheep. Animal.

[ref-14] Dingemanse NJ, Both C, Drent PJ, Van Oers K, Van Noordwijk AJ (2002). Repeatability and heritability of exploratory behaviour in great tits from the wild. Animal Behaviour.

[ref-15] Douglas LA, Varlinskaya EI, Spear LP (2003). Novel-object place conditioning in adolescent and adult male and female rats: effects of social isolation. Physiology & Behavior.

[ref-16] Dwyer C (2004). How has the risk of predation shaped the behavioural responses of sheep to fear and distress?. Animal Welfare.

[ref-17] Fairbanks LA (1993). Risk-taking by juvenile vervet monkeys. Behaviour.

[ref-18] Forkman B, Boissy A, Meunier-Salaün M-C, Canali E, Jones R (2007). A critical review of fear tests used on cattle, pigs, sheep, poultry and horses. Physiology & Behavior.

[ref-19] Fornasieri I, Roeder J, Anderson J (1990). Reactions to mirror in three species of lemurs (Lemur fulvus, L. macaco, L. catta). Comptes Rendus de l’Academie des Sciences Serie III, Sciences de la vie.

[ref-20] Fox RA, Millam JR (2004). The effect of early environment on neophobia in orange-winged Amazon parrots (Amazona amazonica). Applied Animal Behaviour Science.

[ref-21] Fox RA, Millam JR (2007). Novelty and individual differences influence neophobia in orange-winged Amazon parrots (Amazona amazonica). Applied Animal Behaviour Science.

[ref-22] Franks B, Reiss D, Cole P, Friedrich V, Thompson N, Higgins ET (2013). Predicting how individuals approach enrichment: regulatory focus in cotton-top tamarins (Sanguinus oedipus). Zoo Biology.

[ref-23] Frost AJ, Winrow-Giffen A, Ashley PJ, Sneddon LU (2007). Plasticity in animal personality traits: does prior experience alter the degree of boldness?. Proceedings of the Royal Society of London B: Biological Sciences.

[ref-24] Garnett S, Franklin D (2014). Climate change adaptation plan for Australian birds.

[ref-25] Geisbauer G, Griebel U, Schmid A, Timney B (2004). Brightness discrimination and neutral point testing in the horse. Canadian Journal of Zoology.

[ref-26] Glickman SE, Sroges RW (1966). Curiosity in zoo animals. Behaviour.

[ref-27] Goodrick CL (1973). Exploration activity and emotionality of albino and pigmented mice: inheritance and effects of test illumination. Journal of Comparative and Physiological Psychology.

[ref-28] Gould L, Sussman RW, Sauther ML (1999). Natural disasters and primate populations: the effects of a 2-year drought on a naturally occurring population of ring-tailed lemurs (*Lemur catta*) in southwestern Madagascar. International Journal of Primatology.

[ref-29] Gray JA (1987). The psychology of fear and stress.

[ref-30] Hampy DB (1978). Home range and seasonal movement of Barbary sheep in the Palo Duro Canyon. Doctoral dissertation.

[ref-31] Heyser CJ, Chemero A (2012). Novel object exploration in mice: not all objects are created equal. Behavioural Processes.

[ref-32] Inoue-Nakamura N (1997). Mirror self-recognition in nonhuman primates: a phylogenetic approach. Japanese Psychological Research.

[ref-33] Kaczmarek ŁD, Bączkowski B, Enko J, Baran B, Theuns P (2014). Subjective well-being as a mediator for curiosity and depression. Polish Psychological Bulletin.

[ref-34] Kendrick K (2008). Sheep senses, social cognition and capacity for consciousness. The welfare of sheep.

[ref-35] Knudsen EI (2004). Sensitive periods in the development of the brain and behavior. Journal of Cognitive Neuroscience.

[ref-36] Koolhaas J, Korte S, De Boer S, Van Der Vegt B, Van Reenen C, Hopster H, De Jong I, Ruis M, Blokhuis H (1999). Coping styles in animals: current status in behavior and stress-physiology. Neuroscience & Biobehavioral Reviews.

[ref-37] Lilley MK, Kuczaj SA, Yeater DB, Vonk J, Weiss A, Kuczaj SA (2017). Individual differences in nonhuman animals: examining boredom, curiosity, and creativity. Personality in nonhuman animals.

[ref-38] Lopez J, Gómez Y, Rodríguez F, Broglio C, Vargas J, Salas C (2001). Spatial learning in turtles. Animal Cognition.

[ref-39] Maner JK, Gerend MA (2007). Motivationally selective risk judgments: do fear and curiosity boost the boons or the banes?. Organizational Behavior and Human Decision Processes.

[ref-40] Mason GJ (2010). Species differences in responses to captivity: stress, welfare and the comparative method. Trends in Ecology & Evolution.

[ref-41] Mason G, Burn CC, Dallaire JA, Kroshko J, Kinkaid HM, Jeschke JM (2013). Plastic animals in cages: behavioural flexibility and responses to captivity. Animal Behaviour.

[ref-42] McDougall PT, Réale D, Sol D, Reader SM (2006). Wildlife conservation and animal temperament: causes and consequences of evolutionary change for captive, reintroduced, and wild populations. Animal Conservation.

[ref-43] Mehta PH, Gosling SD (2008). Bridging human and animal research: a comparative approach to studies of personality and health. Brain, Behavior, and Immunity.

[ref-44] Mettke-Hofmann C, Winkler H, Leisler B (2002). The significance of ecological factors for exploration and neophobia in parrots. Ethology.

[ref-45] Murphy LB (1978). The practical problems of recognizing and measuring fear and exploration behaviour in the domestic fowl. Animal Behaviour.

[ref-46] Nakamichi M, Koyama N (1997). Social relationships among ring-tailed lemurs (Lemur catta) in two free-ranging troops at Berenty Reserve, Madagascar. International Journal of Primatology.

[ref-47] Napolitano F, De Rosa G, Braghieri A, Grasso F, Bordi A, Wemelsfelder F (2008). The qualitative assessment of responsiveness to environmental challenge in horses and ponies. Applied Animal Behaviour Science.

[ref-48] Oswald ME, Drew RE, Racine M, Murdoch GK, Robison BD (2012). Is behavioral variation along the bold-shy continuum associated with variation in the stress axis in zebrafish?. Physiological and Biochemical Zoology.

[ref-49] Powell DM, Svoke JT (2008). Novel environmental enrichment may provide a tool for rapid assessment of animal personality: a case study with giant pandas (Ailuropoda melanoleuca). Journal of Applied Animal Welfare Science.

[ref-50] Reilly P, Cullen J (1982). The little Penguin Eudyptula minor in Victoria III. Dispersal of chicks and survival after banding. Emu-Austral Ornithology.

[ref-51] Richter SH, Garner JP, Würbel H (2009). Environmental standardization: cure or cause of poor reproducibility in animal experiments?. Nature Methods.

[ref-52] Rilling JK, Barks SK, Parr LA, Preuss TM, Faber TL, Pagnoni G, Bremner JD, Votaw JR (2007). A comparison of resting-state brain activity in humans and chimpanzees. Proceedings of the National Academy of Sciences of the United States of America.

[ref-53] Sharman G, Pilton PE (1964). The life history and reproduction of the red kangaroo (Megaleia rufa). Proceedings of the Zoological Society of London.

[ref-54] Sivak J, Millodot M (1977). Optical performance of the penguin eye in air and water. Journal of Comparative Physiology.

[ref-55] Stahel C, Gales R, Burrell J (1987). Little penguin: fairy penguins in Australia.

[ref-56] Stoinski TS, Jaicks HF, Drayton LA (2012). Visitor effects on the behavior of captive western lowland gorillas: the importance of individual differences in examining welfare. Zoo Biology.

[ref-57] Stöwe M, Bugnyar T, Loretto M-C, Schloegl C, Range F, Kotrschal K (2006). Novel object exploration in ravens (Corvus corax): effects of social relationships. Behavioural Processes.

[ref-58] Svartberg K (2005). A comparison of behaviour in test and in everyday life: evidence of three consistent boldness-related personality traits in dogs. Applied Animal Behaviour Science.

[ref-59] Tetley C, O’Hara S (2012). Ratings of animal personality as a tool for improving the breeding, management and welfare of zoo mammals. Animal Welfare-The UFAW Journal.

[ref-60] Underwood R (1982a). On surveying ungulate groups. African Journal of Ecology.

[ref-61] Underwood R (1982b). Vigilance behaviour in grazing African antelopes. Behaviour.

[ref-62] Wang H, Li J (2015). How trait curiosity influences psychological well-being and emotional exhaustion: the mediating role of personal initiative. Personality and Individual Differences.

[ref-63] Watters JV, Powell DM (2012). Measuring animal personality for use in population management in zoos: suggested methods and rationale. Zoo Biology.

[ref-64] Wilkinson A, Chan H-M, Hall G (2007). Spatial learning and memory in the tortoise (Geochelone carbonaria). Journal of Comparative Psychology.

[ref-65] Wilkinson A, Kuenstner K, Mueller J, Huber L (2010a). Social learning in a non-social reptile (Geochelone carbonaria). Biology Letters.

[ref-66] Wilkinson A, Mandl I, Bugnyar T, Huber L (2010b). Gaze following in the red-footed tortoise (Geochelone carbonaria). Animal Cognition.

