# Supplementary figures and images for: Curious creatures: a multi-taxa investigation of responses to novelty in a zoo environment
Source: PeerJ. 2018 Mar 8;6:e4454. doi: 10.7717/peerj.4454 (PMC5845565; doi:10.7717/peerj.4454)

a)
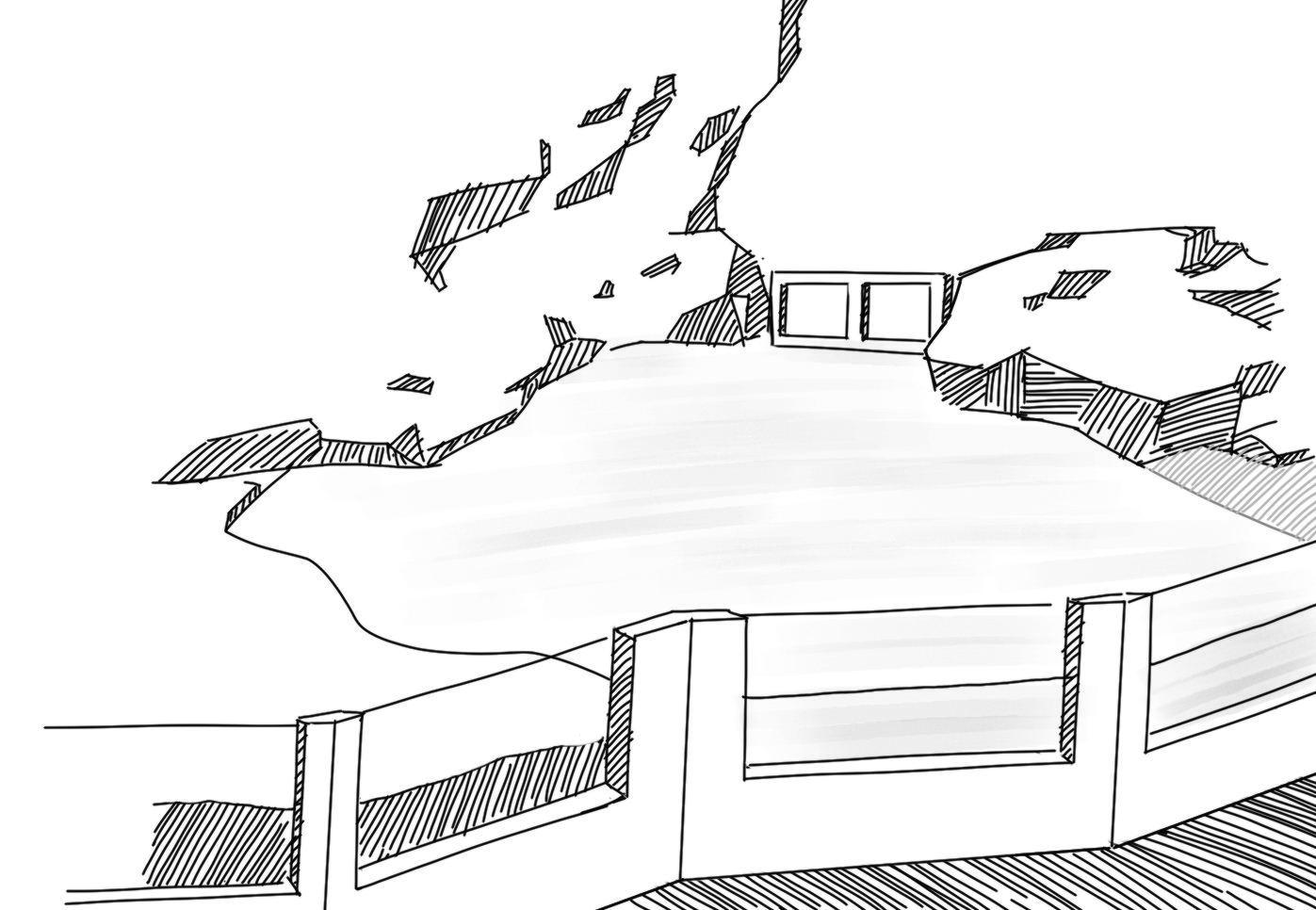


X

M

S


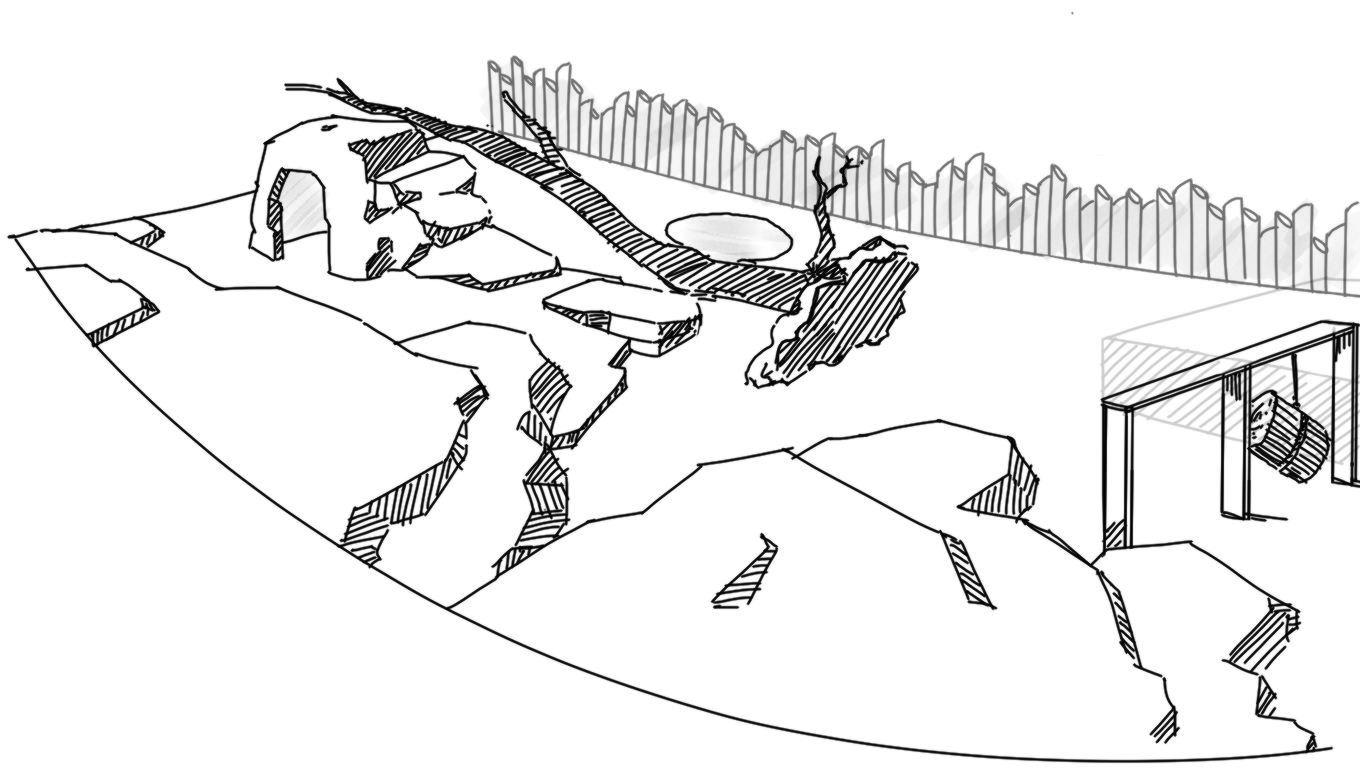


M

X

S

b)

c)
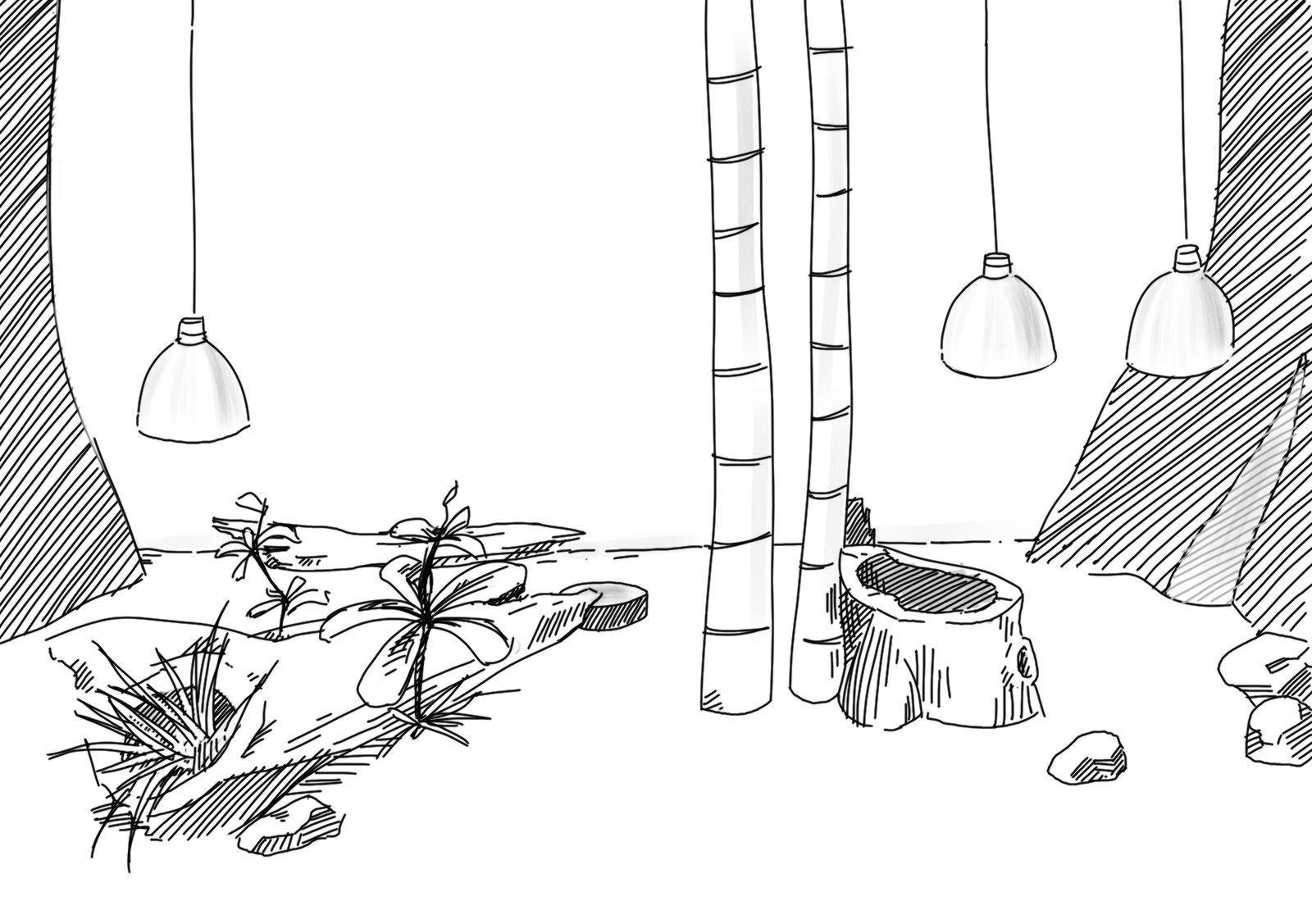


M

X

S

d)
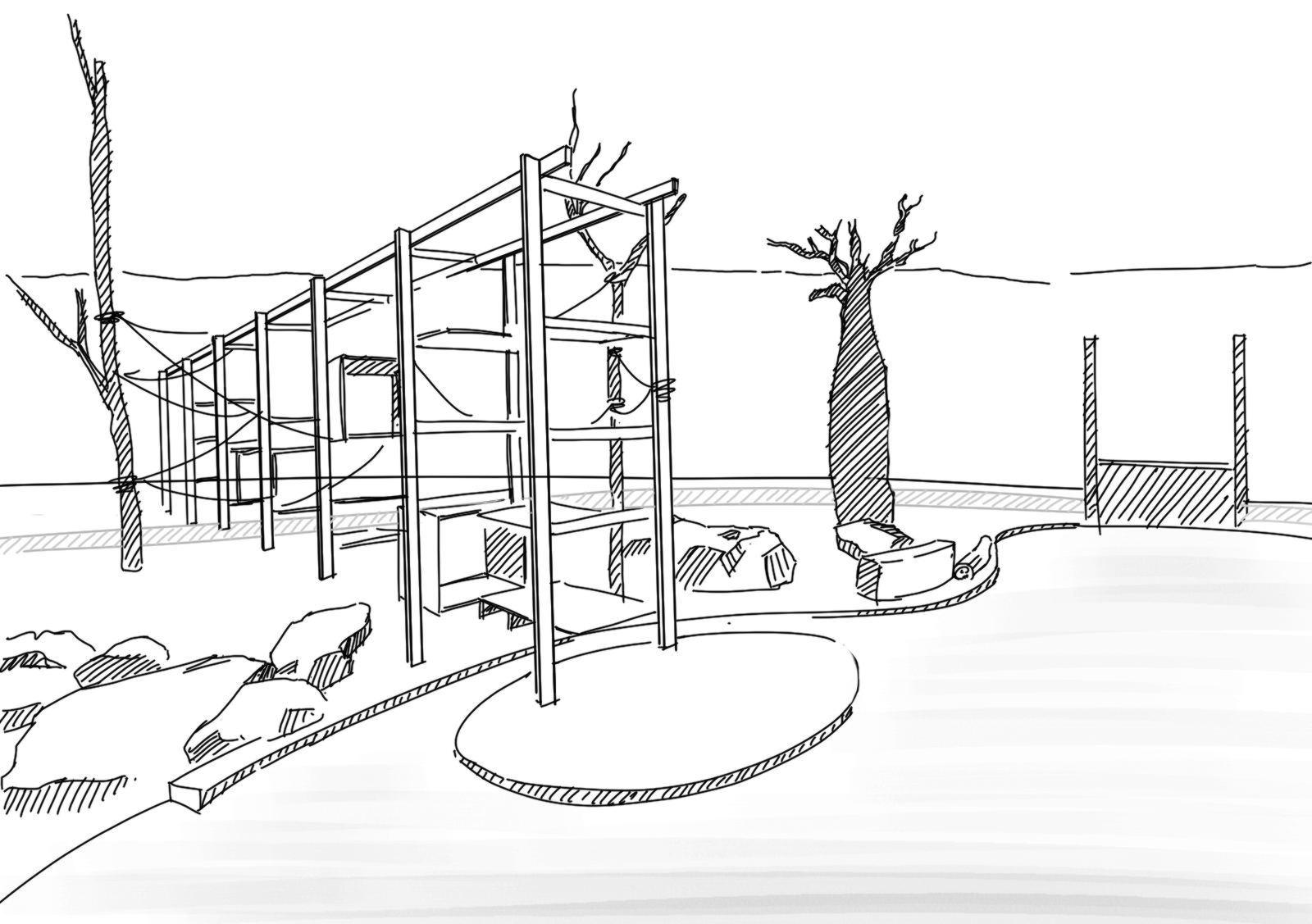


S

X

M

e)
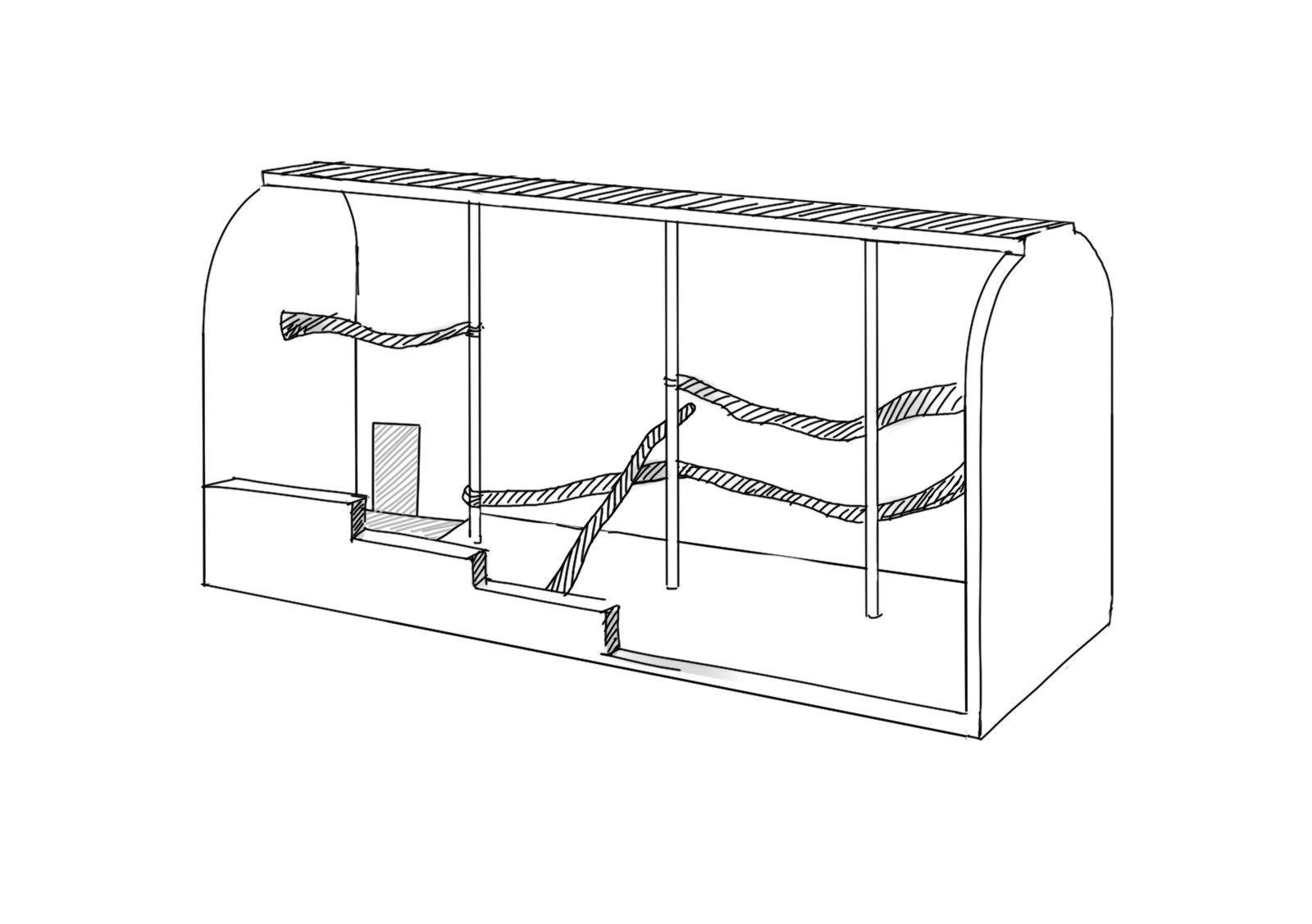


S

X

M


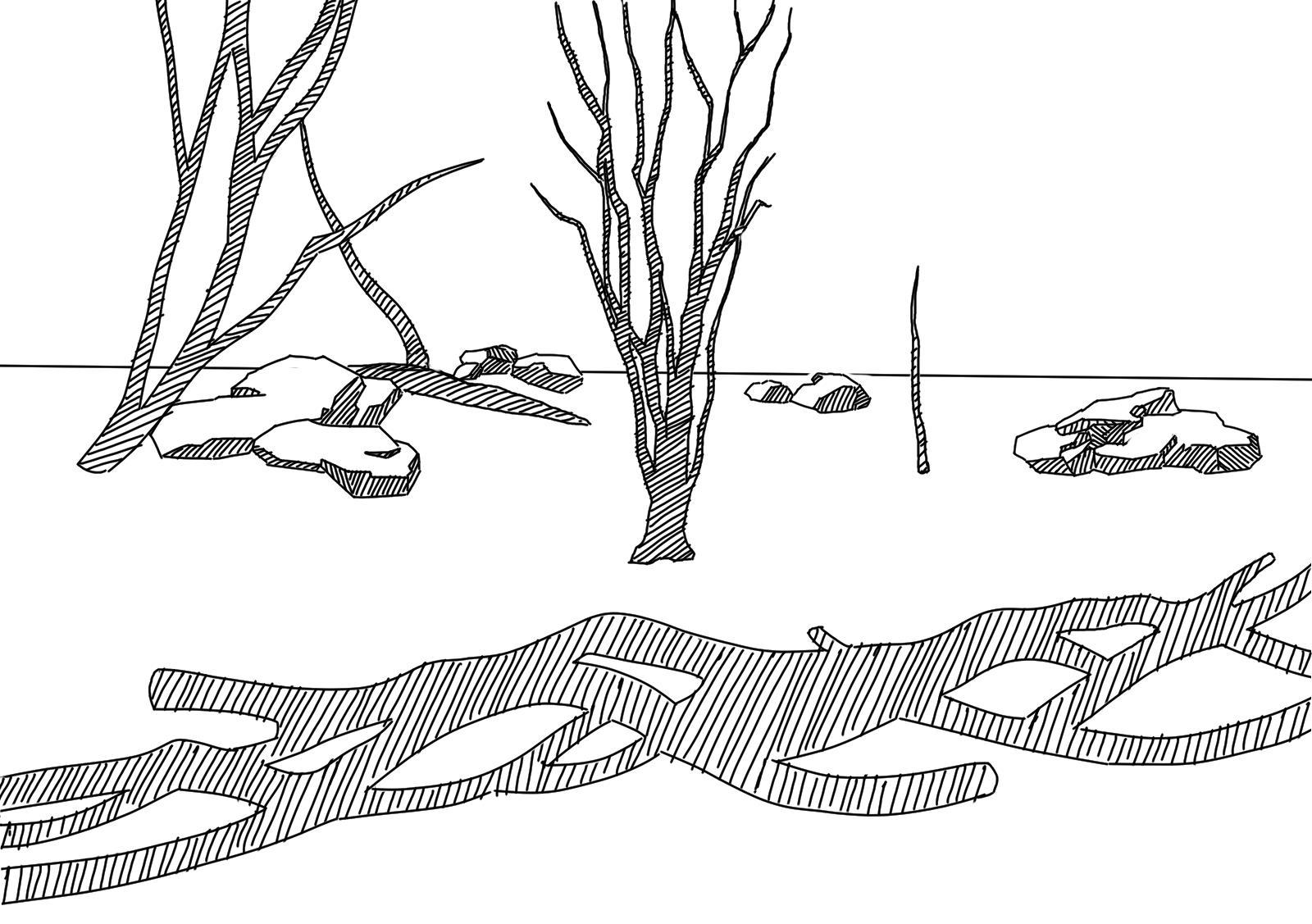


X

M

S

f)

Supplement: Supplemental Information 2 — Layouts of enclosures and novel object placement. Objects identified as moving (M), stationary (S) and mirror (X). a) little penguins b) Barbary sheep c) star tortoises d) ring tailed lemurs e)red-tailed black cockatoos f) red kangaroos. [file peerj-06-4454-s002.docx]
